# Sexual reproduction and auxospore development in the diatom *Biddulphia biddulphiana*

**DOI:** 10.1371/journal.pone.0272778

**Published:** 2022-09-06

**Authors:** Irena Kaczmarska, James M. Ehrman, Matt P. Ashworth

**Affiliations:** 1 Department of Biology, Mount Allison University, Sackville, New Brunswick, Canada; 2 Digital Microscopy Facility, Mount Allison University, Sackville, New Brunswick, Canada; 3 Department of Molecular Biosciences, University of Texas at Austin, Austin, Texas, United States of America; Shiv Nadar University, INDIA

## Abstract

Phylogenetic relationships among mediophycean diatoms with elliptical valve outline and elevated apices have long been a subject of interest and debate, particularly with respect to their relationship to pennates. However, results remain inconclusive, whether based on vegetative valve morphology, reproduction, or molecular phylogenetic data. Searching for phylogenetically informative features, we re-examined sexual reproduction, auxospore structure and development in the diatom *Biddulphia biddulphiana*. Several unique or unusual features and processes characterized its sexual reproduction. A unique spermatogenesis occurs with premeiotic separation of an anucleate protoplast containing all chloroplasts and likely other organelles. Additionally, their auxospore walls are some of the most complex documented, retaining earlier deposited layers that obscure layers formed during later stages of development. The oldest layer consists of thick, mostly organic incunabulum, underlain by outer and inner epizonia and finally transverse (TP) and longitudinal (LP) perizonia. The complexity of the fine structure of these layers is unprecedented. The orientation of some TP bands is also unique in mediophytes, with some perpendicular to the auxospore apical axis, parallel to each other, and open with aligned ends, as typically seen in pennates. The TP also contains rings slanting toward the apices, as in some other mediophytes, e.g., eupodiscaceans. However, both eupodiscaceans and biddulphiaceans show perizonial band structure derived from anastomosing radial scales, thus termed “scaly bands”. Pinnate TP bands, common among pennate auxospores, were not found. Thus *B*. *biddulphiana* auxospore wall structure contains a mixture of characters specific to this clone but also known from mediophytes and araphid pennates. However, these features do not provide unequivocal evidence that this or the other *Biddulphia* species examined to date are the closest extant relatives of basal araphid pennates.

## Introduction

The diatom class Mediophyceae contains a number of genera characterized by elliptical valve outline, radial valve patterning, and fields of pores on elevated apices. These genera have a complicated history of taxonomic affiliations, as the list of their synonyms attests [1]. As a group, they have long been of interest due to earlier work suggesting that they may harbor the most recent common ancestor with the pennates [2, 3]. Despite progress, both classical [4, 5] and molecular [3, 6] work have left some long-standing questions regarding their taxonomic and phylogenetic affinities unresolved. This is also true for the diatom clone investigated here, *Biddulphia biddulphiana* (J.E. Smith) Boyer.

At least twelve mediophycean species with elongated ellipsoid valves have been sexualized and their sex-cells examined using light and/or electron microscopy to date. Reproductive characters considered included sexual behavior and auxospore fine structure [2, 7–16]. Some of these reproductive characters correspond with valve morphology in some species (such as the type of incunabula to the type of apical pore fields). These characters might be used to reorganize taxa within and between families in the Mediophyceae, but no clear trends in such diagnostic reproductive characters have been identified. Nevertheless, there is significant potential for such characters among the Mediophyceae, as the morphological and genetic diversity found in this class is much higher than suggested by the taxonomy within. For example, based on electron microscopy, species once attributed to the genus *Biddulphia* have since been transferred into at least four genera and two different families with different types of cross-sectional ultrastructure of the undulate valve face and elevated apical pore fields which have traditionally defined the genus [4, 6].

Among the taxonomic transfers indicated above, ten species that have had their sexual reproduction examined now belong to the genera *Odontella*, *Trieres*, *Cerataulus*, *Pleurosira*, and *Hobaniella*. They are now included into the monophyletic family Eupodiscaceae [4, 6, 17, 18], characterized by hyaline rims surrounding their elevated apical pore fields (“ocelli”). In contrast, sexuality and auxospore structure has been examined in only two species remaining in the genus *Biddulphia*, characterized by elevated apical pore fields defined solely by pore size (“pseudocelli”), and are currently included in the broadly defined, paraphyletic family Biddulphiaceae. These two species are *B*. *biddulphiana* (as *B*. *pulchella* S.F. Gray in [2]) and *B*. *tridens* (Ehrenberg) Ehrenberg (Samanta et al. 2020).

Despite considerable interest and effort, the complex and robust nature of the siliceous elements in *Biddulphia* auxospore walls made it impossible to untangle many structural details using methods available in early 1980s [2]. The author wrote that this was regrettable because “the auxospore points to the Biddulphiaceae as a family from which pennates could have evolved. This conclusion is somewhat at odds with the analysis by Simonsen [5] based on vegetative valve structure and morphology of living and fossil diatoms that converges on his family Eupodiscaceae, in which *Odontella* and *Triceratium* belong” ([2]; p. 147). Specifically, in 1982 he suggested that the structure and position of the bands of the transverse perizonium (TP) relative to the apical axis of the initial frustule may be phylogenetically informative and should be investigated in comparison to those of araphid pennates. Regrettably, at the time, the fine structure of the auxospore walls was known only for two araphid pennate species of *Rhabdonema*. However, current molecular phylogenetics suggest that this genus is likely derived within the araphids rather than near the base of the pennate divergence [19, 20]; the so-called “core” and “basal” araphids, respectively.

Both the uncertainties and interests presented above warrant re-investigation of the sexual reproduction, auxospore development and structure in a member of the broadly defined, and paraphyletic family Biddulphiaceae, *B*. *biddulphiana*, using currently available technologies. We will focus on the genesis, structure, and development of sex cells and compare them to those found in the basal araphid pennates. Moreover, because valve morphology in the diatom reported as *Biddulphia biddulphiana* includes at least two distinguishable morphotypes (compare [21] to [22]; both as *B*. *pulchella*) we clearly identify the type examined here as the morphotype with oblong valves bearing crenate margins, one or more annulus in postsexual cells, and no processes.

## Materials and methods

### Clone origin and characterization

*Biddulphia biddulphiana* clone HK328 (= ECT3902) was isolated as a single cell from material collected from Florida Bay, Florida, USA (24°50’19.5"N 80°46’22.6"W) in March 2011 and maintained on f/2 growth medium at 20–24°C by MPA. Its DNA sequence data was first published in Ashworth et al. ([6]; nuclear-encoded small subunit ribosomal RNA, chloroplast-encoded *rbc*L and *psb*C markers) while four chloroplast-encoded sequences were added in Theriot et al. ([19]; *atp*B, *psb*A, *psa*A and *psa*B markers). All molecular phylogenetic trees based on this sequence data showed this clone to be sister to another *B*. *biddulphiana* clone (ClayHI, isolated from material collected in Hawaii, USA). The ClayHI clone exhibited all characters seen on valves of ECT3902, plus labiate processes and occluded tubes on the central elevation and a marginal ridge. These two clones were then sister to two clones of *B*. *tridens* ([19]; supplemental fig S2). Here, we use the term “clone” because of its wide understanding, with full appreciation of the potential existence of replication errors and mitotic recombination in cultures grown over years, which as far as documented to date does not equal the magnitude of meiotic gene reshuffling and recombination that takes place during sexual reproduction.

### Growth and sexualization protocol

Prior to induction the ECT3902 stock was grown on the lab bench with an 8:16 h L:D cycle, light intensity ~24 μmol photons^−2^s^−1^ at 22–25°C on f/2 growth medium enriched with silicates (2 ml l^-1^ of f/2) and 27 ppt salinity, using Bay of Fundy seawater.

For induction, cultured cells (~3-week-old stocks) were placed in 6-well mating trays and moved to a growth cabinet at ~16°C, irradiance 25–30 μmol photons^−2^s^−1^ and 12:12 h L:D cycle for 24 h. Then trays were moved back to the lab and inspected for signs of sexual activity. The growth conditions during this time were 22–24°C, continuous light for 3 days with a light intensity of ~24 μmol photons^−2^s^−1^ followed by 8:16 h L:D again for as long as sexualized cells grew. All material was fixed in 2.5% glutaraldehyde in growth media (1:10 vol:vol ratio) at 4°C and stored under refrigeration until prepared for examination.

### Sample processing

#### Light microscopy

To examine the nuclear behavior during various stages of sexual reproduction, cells were stained with Vectashield Mounting Medium with DAPI (4’,6-diamidino-2-phenylindole; Vector Laboratories, Burlingame, CA, USA) as per manufacturer’s instructions. Cell wall silicification was visualized by incorporation of PDMPO (2-(4-pyridyl)-5-((4-(2dimethylaminoethylaminocarbamoyl)methoxy)phenyl)oxazole; Thermo Fisher Scientific, Waltham, MA, USA) into developing cell walls. Silica structures deposited before PDMPO was added to the growth medium are not labeled. A final concentration of 0.125 μM PDMPO was added to culture plate wells prior to or during induction experiments [23]. Brightfield, DAPI, and PDMPO epifluorescence light microscopy were performed using a Zeiss AxioSkop 2 plus upright microscope (Carl Zeiss, Oberkochen, Germany) equipped with an AxioCam HR color camera and HBO 100 mercury vapor or an X-Cite LED (Excelitas Technologies, Waltham, MA, USA) illumination source.

#### Scanning electron microscopy (SEM) and energy dispersive x-ray spectroscopy (EDS)

Vegetative frustules were prepared for SEM/EDS examination following Kaczmarska et al. [24]. Developmental stages with lightly and non-silicified cells were fixed for at least 1 h in 2.5% glutaraldehyde in f/2 media, then rinsed 4X with distilled water (~100 ml) every 10 min with gentle vacuum in a filtration tower onto a 25 mm diameter, 1 μm pore size polycarbonate filter (Sterlitech Corporation, Kent, WA, USA). Filters were removed from the tower while still moist and stacked with stainless steel washers (OD 25 mm, ID 15.5 mm) and an additional filter in the order washer/specimen filter/washer/additional filter/washer and clamped together with a pair of 0.75 in (19 mm) stainless steel fold over binder clips (Staples^®^ Model 24169-CA) to contain the specimen side of the filter in a flow-through chamber. Binder clip handles were removed to allow the assembly to fit into the critical point dryer (CPD) chamber. Specimens were then post-fixed in 1% osmium tetroxide for 1 h, and dehydrated using 10 min changes of 20%, 50%, 70%, 85%, 95% ethanol:distilled water followed by 4 x 10 min changes of 100% anhydrous ethanol. Filter assemblies were dried with liquid CO_2_ in a Denton DCP-1 critical point dryer (Denton Vacuum, Moorestown, NJ, USA).

For examination of more silicified auxospore wall components, specimens were prepared in two ways. In the first method, samples were rinsed of fixative onto filters as above, resuspended in ~1 ml distilled water and subjected to a brief (5 min) acid wash (15 ml 1:1 sulphuric:nitric acid) with no heating other than that produced by mixing of the two acids. The acid solution was then diluted in 100 ml distilled water, transferred to the filtration tower containing a fresh filter, rinsed with 250 ml distilled water and air-dried. The second preparation method omitted the acid treatment; the sample simply was washed with 250 ml distilled water onto the filter and air-dried. Filters bearing specimens prepared by all methods were mounted on aluminum stubs with double-sided tape, rimmed with colloidal carbon, and coated with ca. 15 nm of gold using a Hummer 6.2 sputtering unit (Anatech Ltd., Union City, CA, USA). Images were acquired using a Hitachi SU3500 SEM (Hitachi High Technologies, Toronto, Canada) at a working distance of 5 mm and 10 kV accelerating voltage. EDS was performed with the same instrument equipped with an Oxford AZtec/X-Max 20 EDS system (Oxford Instruments, High Wycombe, UK) at 10 mm working distance. Since the only element of interest in this study was silicon (Si-K_α_, x-ray energy 1.74 keV), an accelerating voltage of 10 kV provided sufficient overvoltage for efficient x-ray excitation. Spectra were acquired for 100 s (dead time corrected) at 0.1 nA beam current, energy range 0–10 keV into 1024 channels. The EDS spectra were collected from intact and unobstructed structures. Spectra from the polycarbonate support filter adjacent to the structures were taken to verify no remote excitation from neighboring siliceous components (if present) at distances as close as 3 μm.

The terminology used here for auxospores, and sexual stages conforms to Kaczmarska et al. [25] while for frustule structure follows Anonymous [26] and Ross et al. [27]. We refer to the internal cross-walls on the vegetative valves as “internal costae” *sensu* Witkowski et al. [28].

## Results

### Parental clone characterization

While in culture, the clone ECT3902 maintained most of the morphological characters of the species as described [29] and illustrated by line drawings and SEM images ([21]; pl. 118, figs 26–32, pl.121, figs 1, 2, [12, 22, 30]). These included the frustule dimensions, elevated pseudocellate apices, central annulus, elaborately branched areolar occlusions, non-occluded accessory pores scattered across the valve face, and internal costae. However, none of our clone specimens exhibited labiate processes or occluded tubes common on the central elevation in specimens of this species shown by Montgomery [31], Hoban [12], Navarro and Lobban [32], Tiffany [30], and many others.

### Sexual reproduction

Sexually competent cells responded to induction cues within a few hours. Valves of sexualized individuals were broadly elliptical to nearly circular in face outline, 25–57 μm in apical axis. Numerous free eggs, open gametangia and first anisodiametric auxospores were observed 12 h after induction. The first full size auxospores were found at the end of the first day after induction, while initial cells and their first vegetative, postsexual division were present a day or two thereafter. The clone also auxosporulated spontaneously, particularly following replenishment of growth media, but resulted in far fewer auxospores and even fewer initial cells. Irrespective of origin, the auxospores were indistinguishable in their structure and development but were distinctly different from vegetatively enlarged cells of this species reported and illustrated by von Stosch ([33]; as *B*. *pulchella*).

### Gametogenesis (Figs 1 and 2)

**Fig 1:**
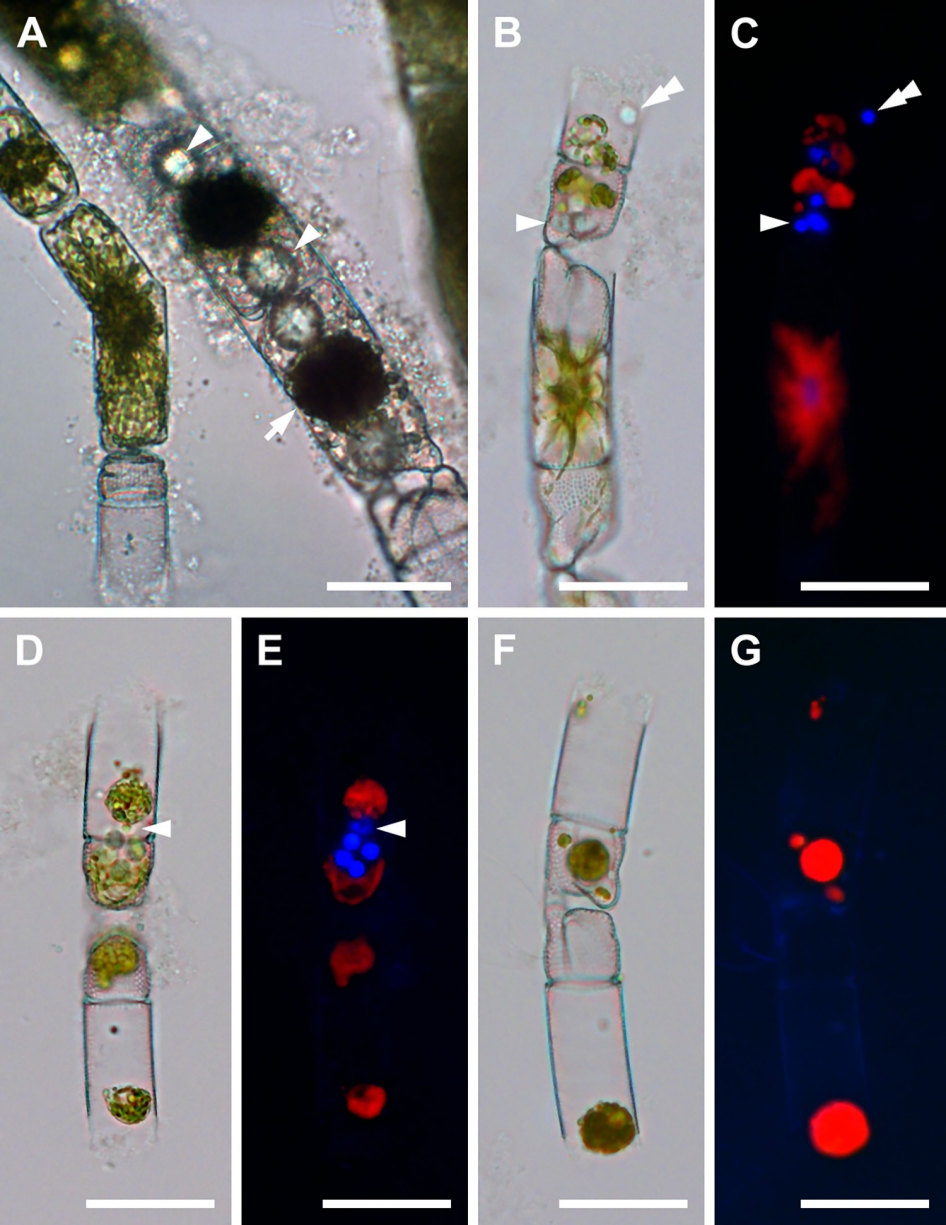
Spermatogenesis (brightfield and DAPI-stained epifluorescence light microscopy). (A) Two centrally located cells each with two spermatocytes (arrowheads) and residual plasmatic bodies with chloroplasts (one indicated by arrow). (B, C) Sexualized male cells, the top theca with two spermatocytes. A tetrad of spermatids from the bottom spermatocyte is indicated by arrowhead (pale spheres in B; DAPI-stained blue nuclei in (C) near the valve face. Top spermatocyte shows only two spermatids remaining within the theca (double arrowhead), residual protoplasts are partially degraded. Red color in all epifluorescence images is due to chlorophyll autofluorescence. (D, E) Two open spermatogonangia. Bottom one contains only remnants of the residual bodies, top theca contains tetrad spermatids plus one stray one (arrowhead) and two residual bodies. (F, G) Thecae of two spermatogonangia, each with residual bodies, similar to the bottom theca shown in (D, E). All scale bars: 50 μm.

Spermatogonia were generally smaller than oogonia. They were 25–43 μm in apical and 83–132 μm in pervalvar axis and each produced at least two naked spermatocyte mother cells (Fig 1A). The spermatocyte mother cells first underwent a separation of the protoplast containing chloroplasts from that with the nucleus, thus resulting in two unequal products. The larger cell contained most of the protoplast and all chloroplasts (a colored mass of 14–41 μm in diameter; Fig 1A, 1F and 1G), a presumed anucleate residual body. The second, smaller cell contained the nucleus and no chloroplasts. It lacked frustules and became a colorless primary spermatocyte (11–23 μm in diameter; Fig 1A). The spermatocytes underwent meiosis, producing four colorless, nonflagellated spermatids; each was 6–10 μm in diameter (Fig 1B–1E). Neither flagellated sperm nor syngamy were observed.

**Fig 2:**
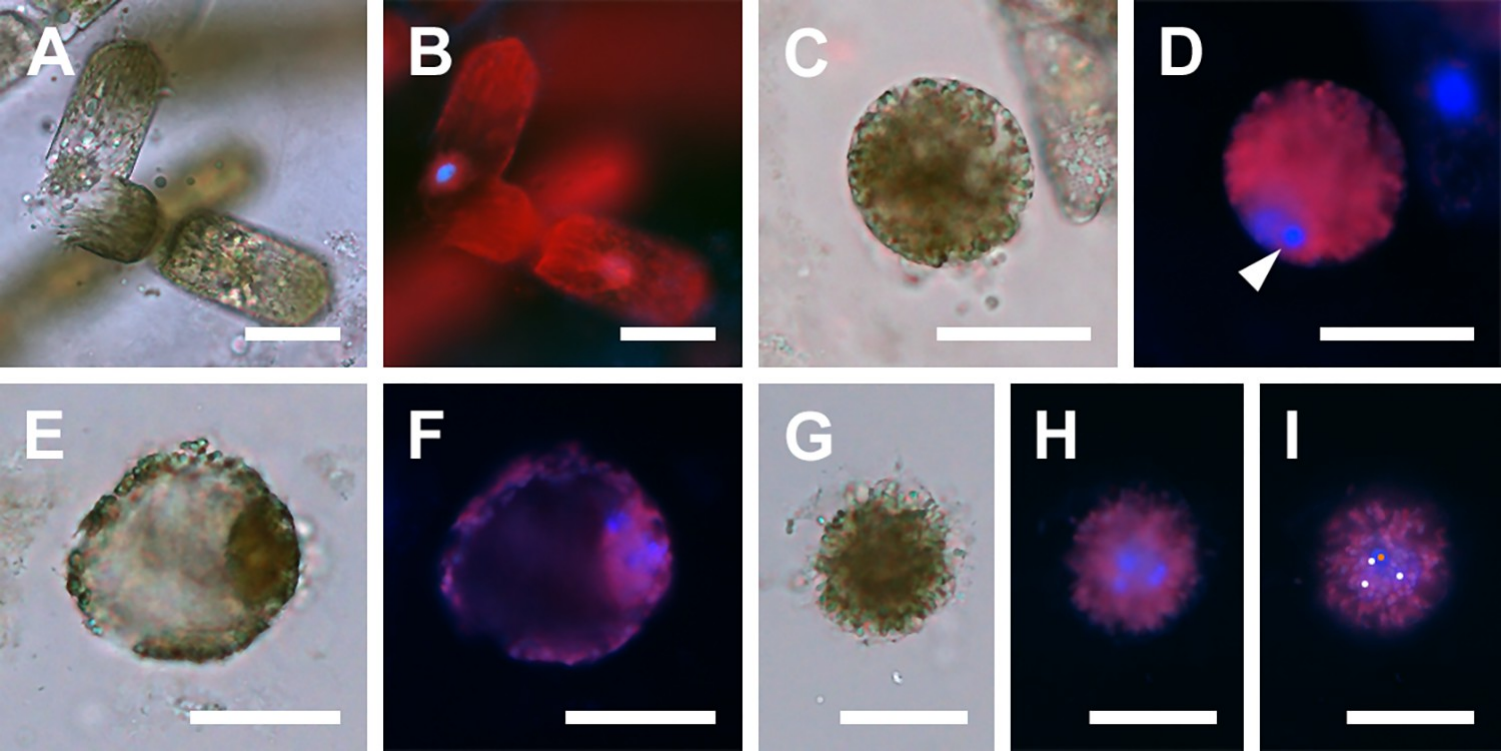
Oogenesis and fertilization (brightfield and DAPI-stained epifluorescence light microscopy). (A, B) Bent oogonium ready to liberate a primary oocyte. (C, D) Free, binucleate secondary oocyte, one nucleus pyknotizing (arrowhead). (E, F) Free, trinucleate diatom egg. Note chloroplasts densely surrounding the nuclei. (G, H, I) Free tetranucleate cell, a putative fertilized diatom egg. (G) Shows condensation of protoplast mass around the nuclei. (H) Shows three nuclei at a common focal plane. (I) Shows the fourth nucleus, at a different focal plane. The centers of the three nuclei shown in (H) are marked by three white dots, the fourth nucleus center is located at a different focal plane and is marked with an orange dot. All scale bars: 50 μm.

Oogonial frustules were generally larger than spermatangial frustules; 40–57 μm in apical axis. One uninucleate oocyte was produced per oogonium (Fig 2A and 2B). After leaving the oogonial frustule, the oocyte became spherical and free-floating in the growth media (Fig 2C–2F). Following acytokinetic meiosis, the free oocytes functioned as eggs, although they could be found with 1–2 pyknotic nuclei in addition to a functional gametic nucleus. It was not always possible to distinguish gametic from pyknotic nuclei (Fig 2F) or ascertain their exact number in all cells due to a great number of autofluorescing chloroplasts that obstructed examination. These free, functional female gametes were recognizable in our material by their dense cell contents, spherical shape, and relatively small size, 59–116 μm in diameter. In some eggs numerous chloroplasts were evenly dispersed, in others some aggregated around the nucleus, with the remainder of the cell containing fewer chloroplasts. The number of nuclei detected by DAPI staining varied from 1–3 in all but one cell. That cell had 4 nuclei and was 71 μm in diameter (Fig 2G–2I). This cell may have represented a fertilized egg at a stage following cytogeny but before karyogamy.

### Spherical and small anisodiametric auxospores (Fig 3)

**Fig 3:**
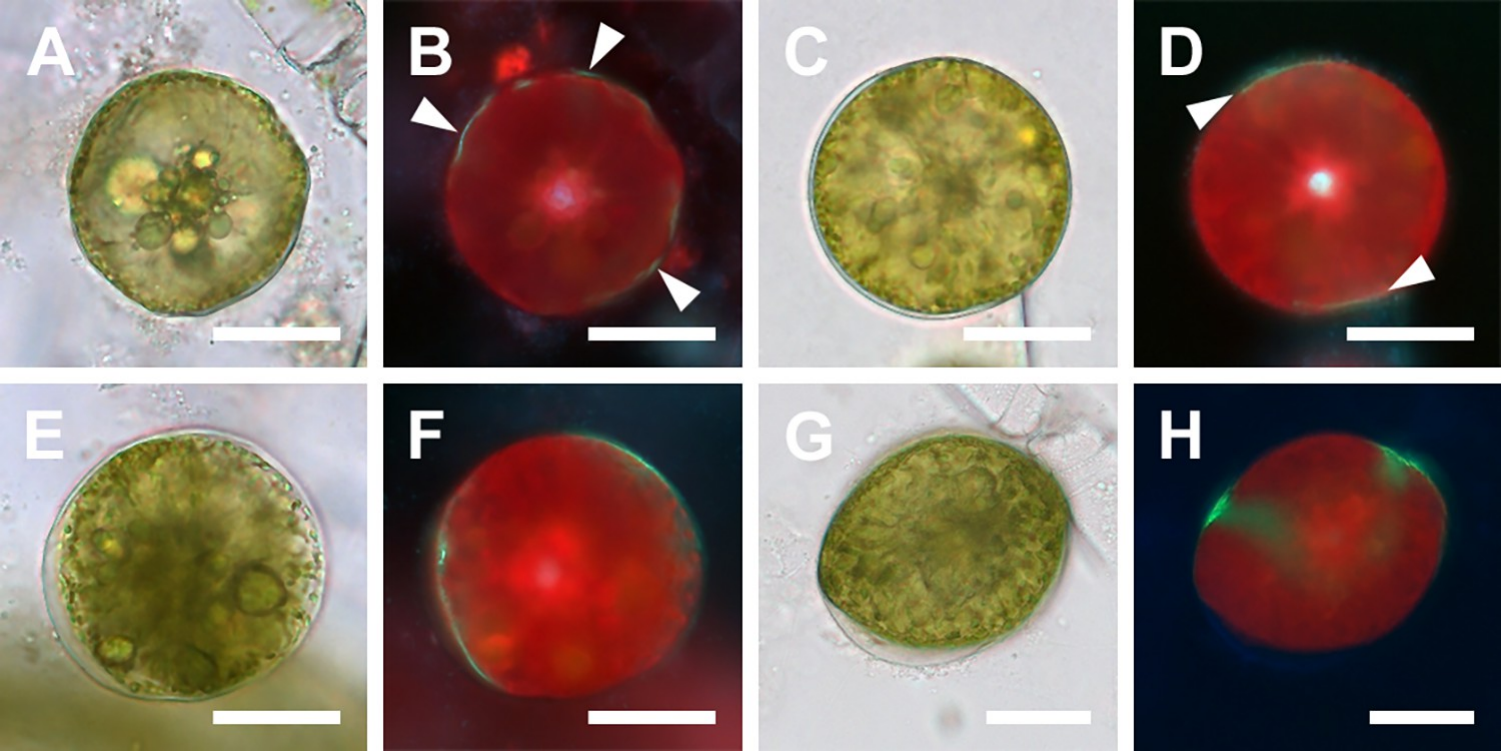
Zygotes and spherical auxospores (brightfield and PDMPO-labeled epifluorescence light microscopy). (A, B) Zygote or young spherical auxospore showing several islands of concurrent silicification of the cell wall (arrowheads in B), likely containing individual or clusters of siliceous scales. (C, D) Spherical auxospore showing deposition of silica in the equatorial region (arrowheads in D). (E, F) An auxospore showing more siliceous elements being deposited. Note the large area of silicification on the protoplast surface pulling away from the older part of the wall, left side. (G, H) A slightly ellipsoidal auxospore showing deposition of two epizonial bands, one arching towards the apex. All scale bars: 50 μm.

The first clear PDMPO-based evidence of deposition of siliceous elements was observed in spherical cells with one large, centrally located nucleus. These cells were 90–110 μm in diameter (Fig 3A–3D), and so generally larger than eggs or the putative tetranucleate spherical zygote shown in Fig 2G–2I. Note that the illustrated cells show only newly deposited silica. In many cases when exposure to PDMPO was relatively short (12–24 hours), the areas of PDMPO-tracked incorporation of silica indicated that its deposition was neither uniform nor directional. Small, slightly anisodiametric auxospores also showed walls or their components incorporating silica (traced by PDMPO). The smallest such cells found were 74–129 μm in long axis and 72–121 μm in short axis; one such cell is illustrated in Fig 3E and 3F. In another subspherical anisodiametric auxospore (130 μm in long and 108 μm in short axis) the first bands had just been deposited (Fig 3G and 3H). Some spherical and anisodiametric auxospores were found swathed with translucent halos lightly tracked with PDMPO (Fig 3F and 3H).

### Larger anisodiametric and mature auxospores (Figs 4 and 5)

**Fig 4:**
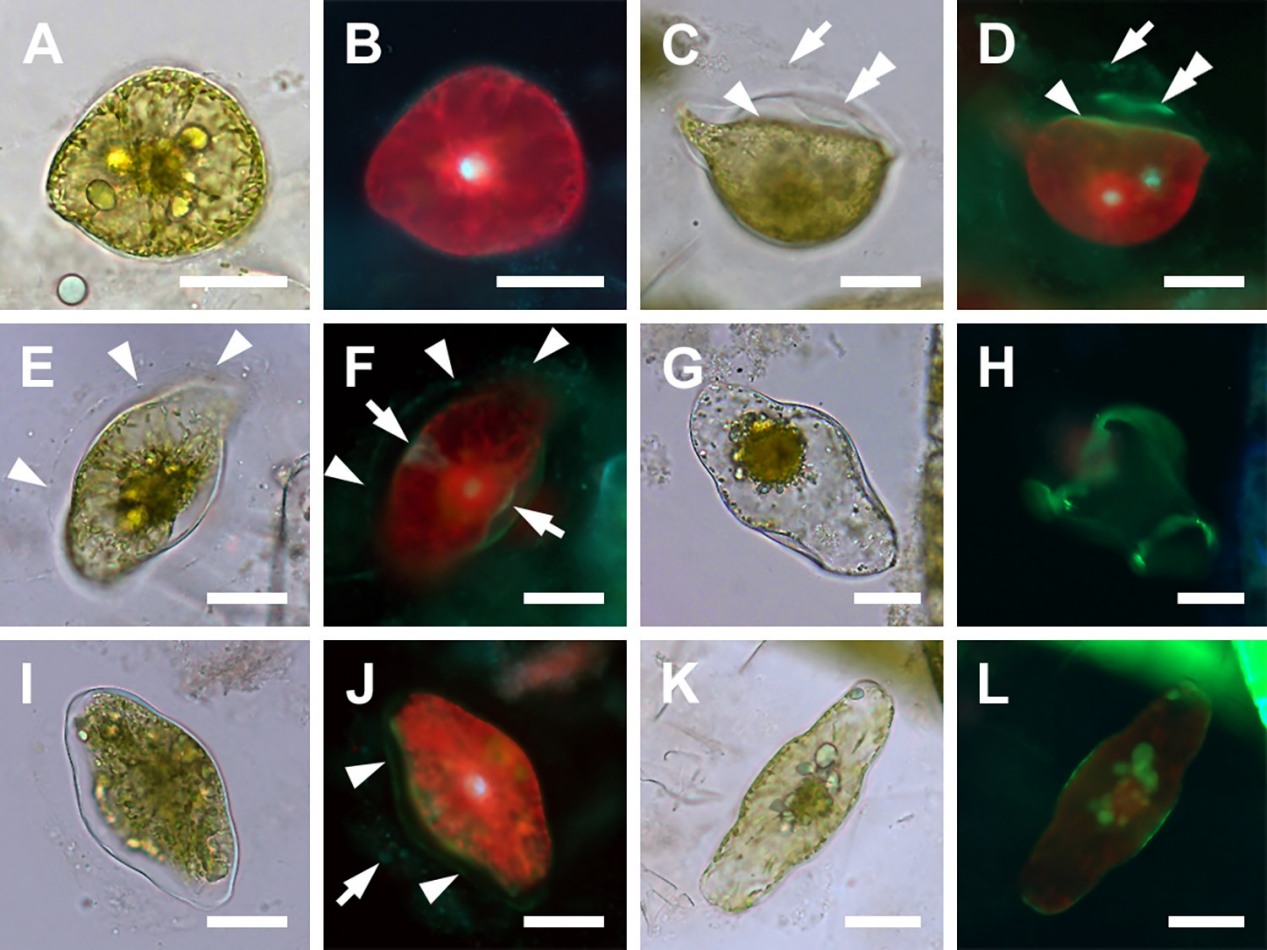
Immature isodiametric auxospores (brightfield and DAPI/PDMPO epifluorescence light microscopy). (A, B) Early isodiametric auxospore showing beginning of first apical bud expansion and faint halo of earlier deposited silica around periphery. (C, D) Girdle view of auxospore with a hemispherical ventral (bottom) side and flattened dorsal (upper) side. Note different size of “apical buds” and three layers of green fluorophore in D indicating successive layers of silica deposition. The oldest represents incunabula (arrow), the next below either a more strongly silicified incunabula or a part of the outer epizonium (double arrowhead), and the layer resting on the cell surface represents remaining epizonium (arrowhead). (E, F) Older auxospore enveloped in translucent incunabula (arrowheads) with some of the later deposited silica best seen above retracting protoplast on the dorsal (right) side. The band circling the mid-cell (arrows in F) likely represents the primary band of TP. (G, H) Aborted and disintegrating auxospore with two PDMPO-labeled open and aligned but not meeting bands of the TP. (I, J) Anisodiametric auxospore with green multilayered wall components, (arrowheads and arrow). (K, L) An oblong auxospore showing concurrent apical and mid-cell areas of silicification. All scale bars: 50 μm.

Transformation of the spherical auxospore into a mature one capable of production of the polar initial frustule involved sequential expansion and localized retraction of the protoplast with concurrent deposition of silica (Fig 4A, 4C, 4E and 4I). As in spherical auxospores, the siliceous wall-elements of larger anisodiametric auxospores were also deposited non-synchronously, asymmetrically, and unevenly, on newly exposed protoplast surfaces (Fig 4C–4F, 4I and 4J), thus physically distant from previously deposited layers. Such a growth pattern in free cells led to recovering different auxospore outlines depending on orientation of the auxospore in the microscope preparation and the stage of silicification in that particular area. For example, while developing wedge-shaped apices (usually one earlier than the other), the same auxospore (Fig 4A–4D) would appear teardrop-shaped in outline if observed in dorsal view, spherical or ellipsoidal if facing either apex, or hemispherical if in many oblique orientations that did not reveal dorsal apices. Initially growing apices took the form of slim cones (Fig 4C and 4D), but later expanded at the bases resulting in lozenge-shaped cells (Fig 4E, 4F, 4I and 4J).

**Fig 5:**
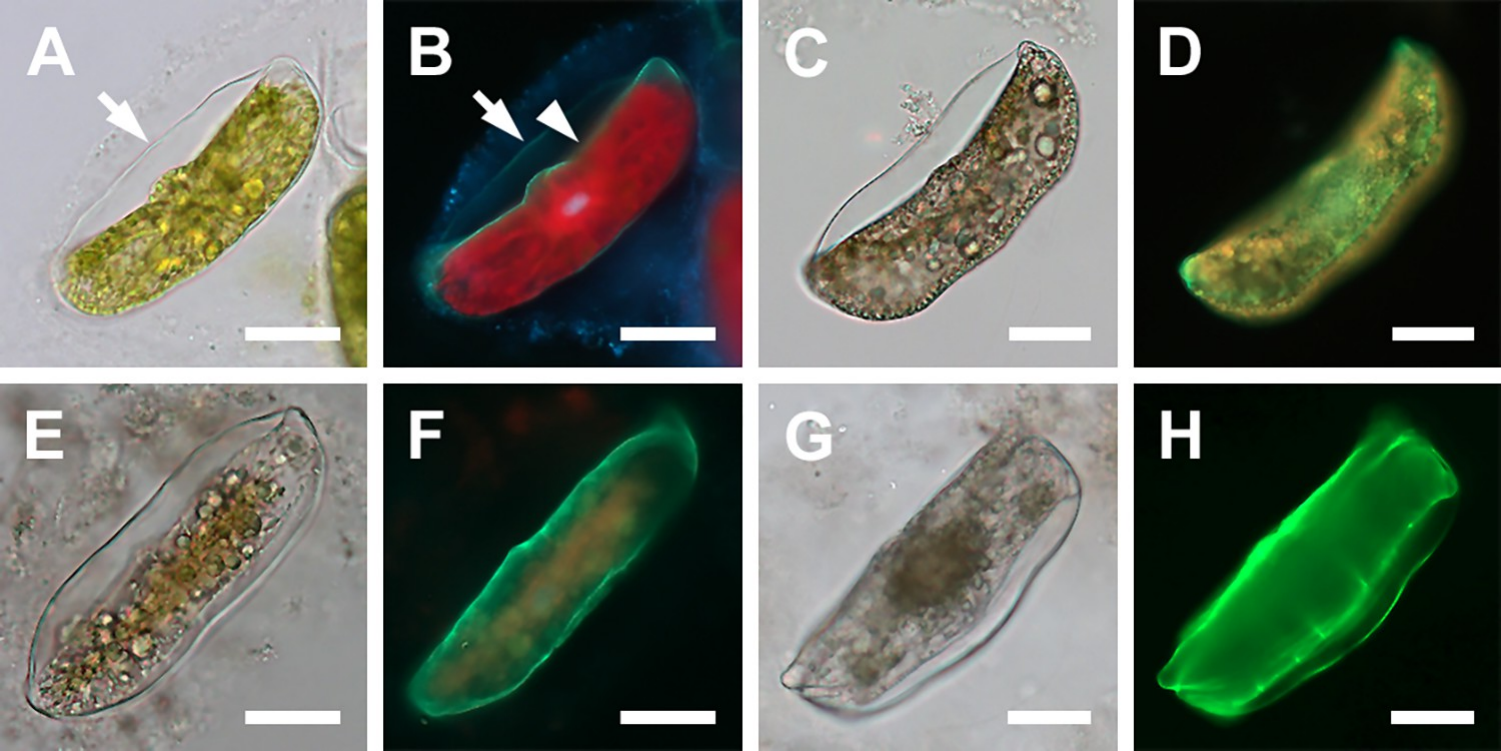
Mature auxospores and deposition of initial valves (brightfield and DAPI/PDMPO epifluorescence light microscopy). (A, B) Mature auxospore in girdle view showing deposition of initial epivalve (arrowhead in B) underneath siliceous wall elements deposited earlier (arrow). (C, D) Mature auxospore in girdle view with more developed initial epivalve, PDMPO-labeled green in (D). Note the absence of internal costae. (E, F) Valve face view of auxospore with initial cell protoplast retracted along both sides of auxospore. (G, H) Mature auxospore with initial frustule in girdle view with the remnants of auxospore wall over the initial hypotheca with internal costae. All scale bars: 50 μm.

Mature auxospores emerged following rounds of deposition of final silica bands primarily near the apices and cell mid-section (Fig 4G, 4H, 4K and 4L). Wall elements laid down later were deposited underneath retained earlier deposited elements, all together enveloping the protoplast in complex, multilayered walls. When more than one silica-containing layer was traced, the younger layers show greater intensity of PDMPO incorporation than the previous layers (Figs 4B, 4D, 4F, 4H, 5A and 5B), suggesting a heavier degree of silicification. As the apical length of the auxospore grew, its transapical length diminished. In spherical/ellipsoidal auxospores the apical axes were 107–132 μm while the transapical axis were 71–108 μm. When mature, they measured 151–328 μm in apical and 49–98 μm in transapical axis. Even some of the large auxospore walls retained delicate and translucent halos (Fig 4C and 4E), such as seen in many spherical cells. The most common form of the auxospore was oblong; the largest was 328 μm in apical and 78 μm in transapical axis. In addition, a few auxospores were inflated in the midsection, and occasionally tripolar auxospores were also found. These were 94–300 μm long along the side available for measurements.

### Deposition of initial frustules (Fig 5)

Both initial valves developed under the previously laid down auxospore wall layers and following protoplast retraction (Fig 5A, 5B, 5G and 5H; initial epivalve and initial hypovalve, respectively). Typically, initial valves were several times larger than the parental valves (161–300 μm compared to 25–57 μm in apical length, respectively; Fig 5C–5F). They also demonstrated less pronounced valve face topography (Fig 5A and 5C) compared to typical, fully formed vegetative valves. The difference was largely due to the relatively lower central and apical elevations with smaller and/or less differentiated pseudocelli on initial valves. Although internal costae were present on some initial epivalves, they were often greatly misoriented compared to those on typical vegetative valves. Well formed valve face elevations and regular internal costae often first appeared on the initial hypovalve (Fig 5G and 5H). Multilayered accumulation of siliceous elements surrounding the initial valve in Fig 5E and 5F represent its mantle and the cingulae, illustrating the still irregular outline of the initial frustule.

### Auxospore wall composition and fine structure (Figs 6–12)

**Fig 6:**
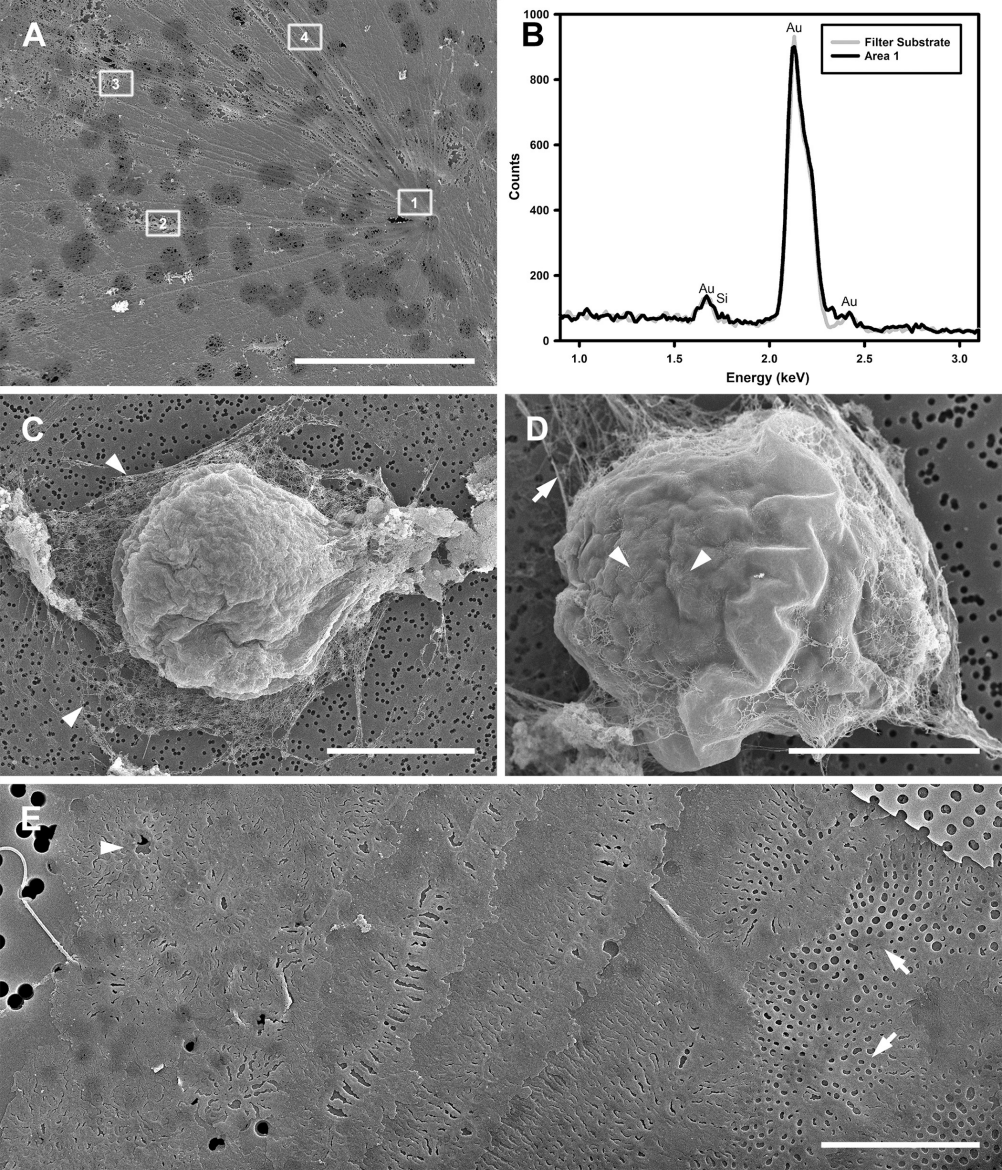
Incunabular elements of the auxospore wall (SEM/EDS, both fixed/critical point dried and acid cleaned specimens). (A, B) Large, organic annular scale, a part of the shroud surrounding auxospores. Framed numbers in (A) indicate where EDS spectra were acquired. (B) Only spectrum from Area 1 is shown compared to spectrum of filter substrate (substrate acquisition area not shown). None of the areas shown here demonstrate the presence of silicon. (C) Egg or zygote swathed in delicate shroud (arrowheads) that was partially damaged and peeled away from cell surface during sample preparation. (D) Spherical auxospore showing remnant of shroud (arrow) and delicate dendritic scales on the auxospore surface (arrowheads). (E) Acid cleaned and partially disarticulated section of the auxospore wall illustrating variety of size, shape and structure of the silicified components ranging from heavily silicified (incunabular?) scales (arrowhead) to epizonial bands and scales (arrows), shown from left to right. Scale bars: A, 10 μm; C, 50 μm; D, 25 μm; E, 10 μm.

**Fig 7:**
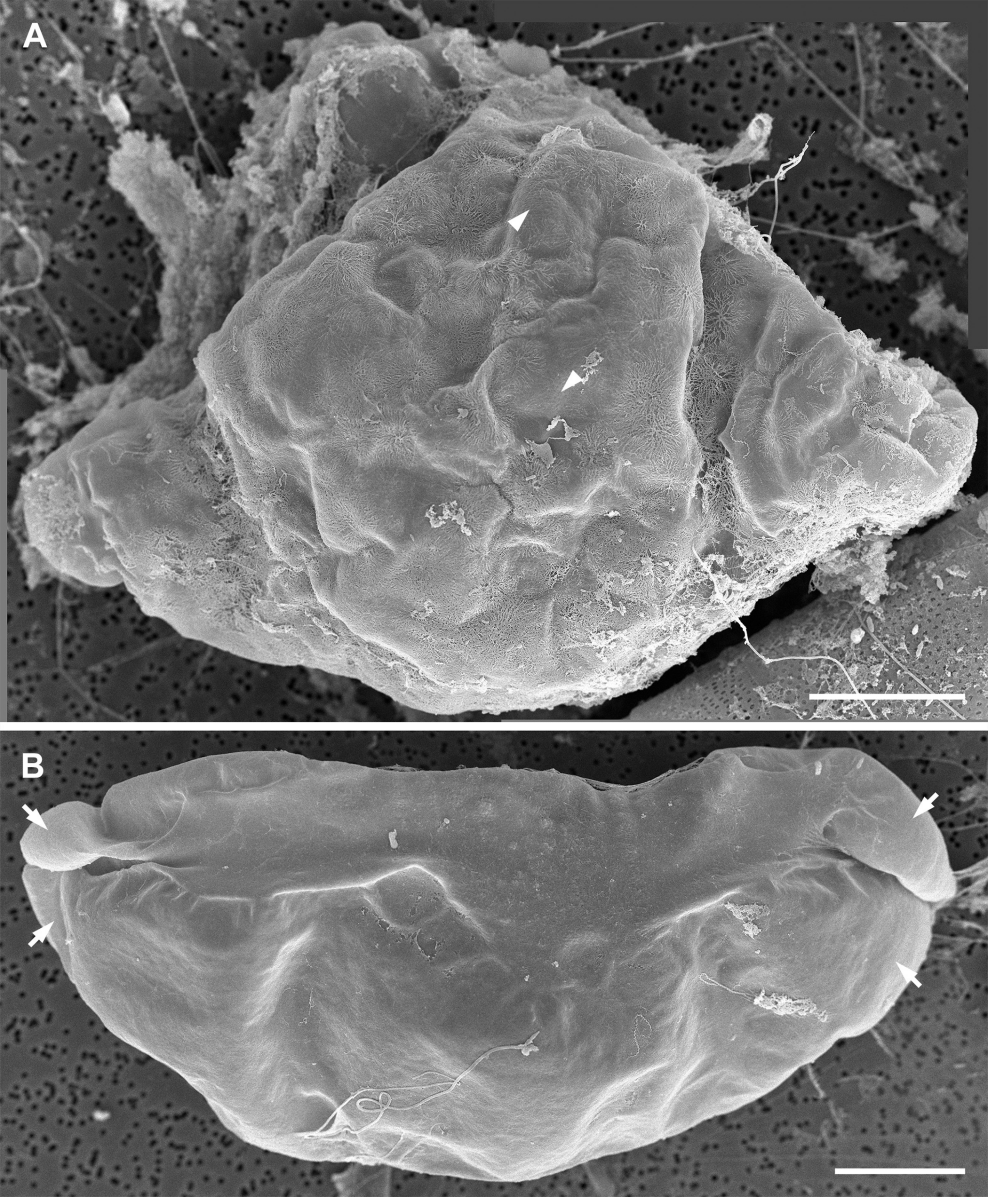
Young anisodiametric auxospores (SEM, fixed/critical point dried specimens). (A) Young auxospore with attenuated apices and dendritic scales on its surface. Note perforation of strongly silicified epizonial elements is best visible in upper right where the older wall elements have thinned (arrowheads). Digital montage of four separate images. (B) Somewhat older auxospore in girdle view with retained 3-dimensional integrity with well developed dorsal (flatter) and ventral (bow-shaped) sides. Sturdy, siliceous elements appearing as well-defined hardened structures at apices (arrows) are presumed to consist of epizonial scales. Digital montage of two separate images. All scale bars: 20 μm.

**Fig 8:**
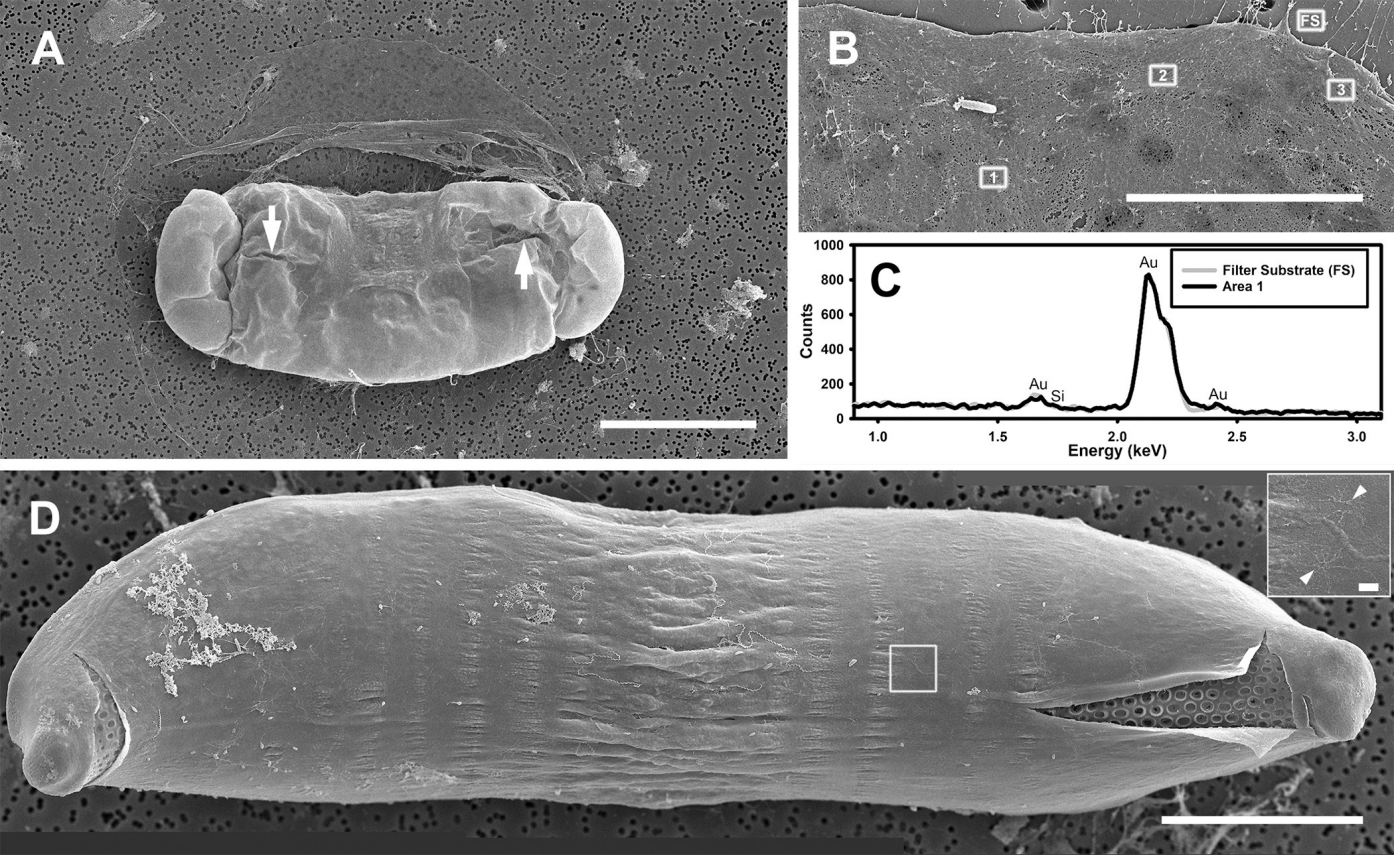
Older and mature auxospores (SEM/EDS, fixed/critical point dried specimens). (A) Ventral side of an oblong auxospore surrounded by remains of delicate shroud. Note rigid siliceous apical caps and gap between open, aligned TP bands (arrows). (B) Fine structure of shroud from (A). Framed numbers and FS indicate where EDS spectra were acquired from shroud and control filter surface. (C) Spectrum from Area 1 compared to spectrum of filter substrate. None of the areas shown here demonstrate presence of silicon. (D) Nearly intact mature auxospore wall enclosing initial valve. Note remains of a few dendritic scales (insert; arrowheads) and parallel ribbing of auxospore wall in midsection. Digital montage of three separate images. Scale bars: A, 50 μm; B, 10 μm; D, 40 μm; D insert, 1 μm.

Critical point dried (CPD) SEM preparations recovered auxospores with walls that retained very delicate, lightly to non-silicified external organic components *in situ*. In these preparations we observed cells in stages comparable to those observed in LM (Fig 4C–4E) but some wall constituents became resolvable and could be individually examined (Figs 6A and 8B). As seen traced by PDMPO, these walls also contained a great number and various types of well silicified elements organized into several different layers. Most of these layers were retained, all thinly stretched and appressed together into a single envelope so that they surrounded the initial frustule even when the auxospore was mature (Fig 8D; note tears at the apices). This retention is evidenced by the presence of dendritic incunabular scales on the wall surface (compare Figs 6D and 7B to Fig 8D insert).

#### Incunabular elements

The most external part of the auxospore wall contained a variety of incunabular elements. They differed in size, shape, structure, and degree of silicification. Some of the elements have not been previously reported.

**Fig 9:**
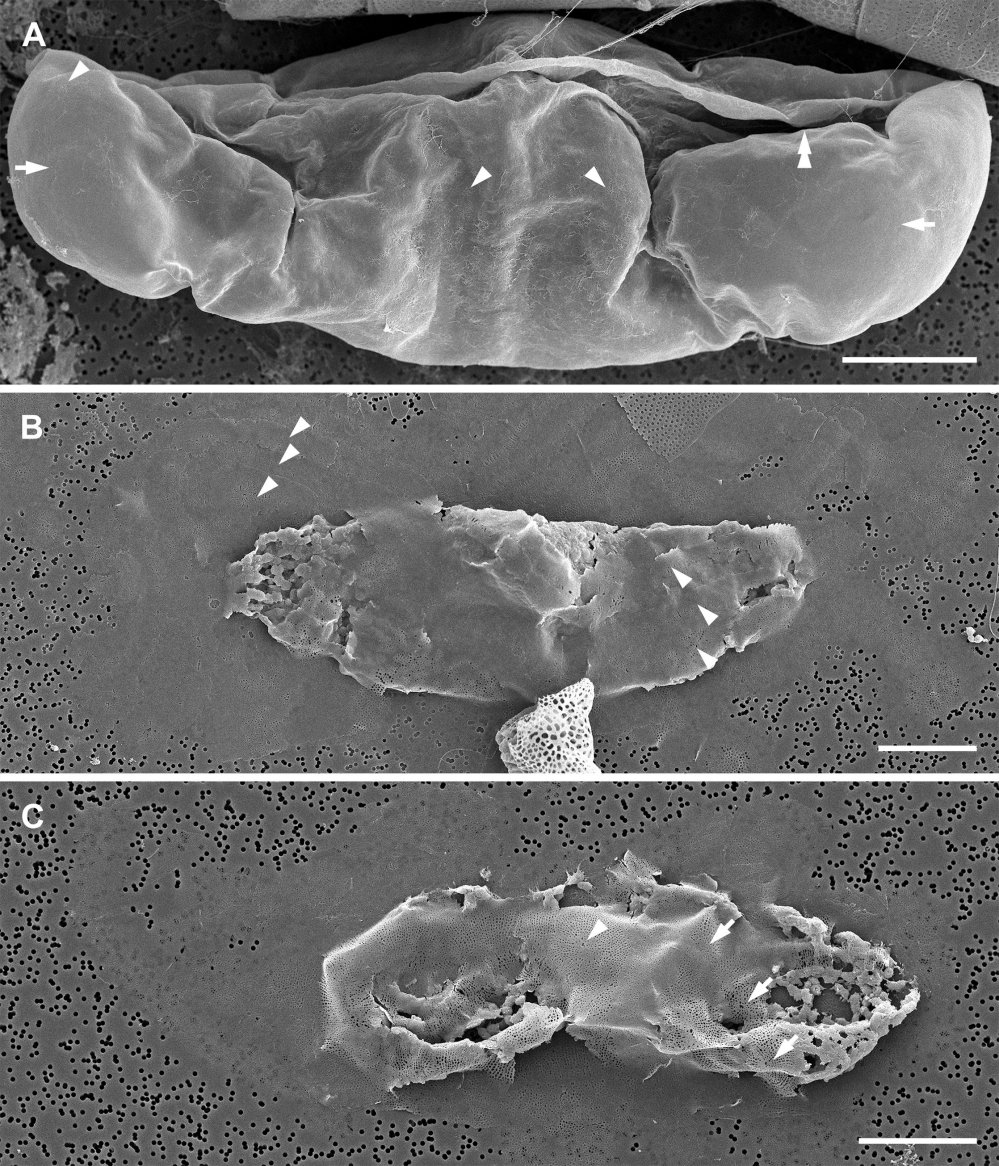
Major layers of the auxospore wall (SEM, both fixed/critical point dried and acid cleaned specimens). (A) Lateral view of a nearly mature auxospore illustrating lasting incunabular cover with dendritic scales (arrowheads), outlines of the apical caps of epizonial scales underneath older wall-layers (arrows), and a gap between TP bands (double arrowhead) at the auxospore venter. (B) Disarticulated outer epizonial layer showing arrangement and structure of the coarse bands (arrowheads) around the cell. (C) Layer of inner epizonial scales (arrowhead) and short bands (arrows) assembled from anastomosing scales. All scale bars: 50 μm.

Spherical cells (eggs, zygotes and auxospores) were enveloped in shrouds (seen as halos in LM images; Figs 6C, 6D and 8A) either with no silica or silicification near the SEM/EDS detection limit. The shrouds contained delicate webs and stronger nets (Figs 6A and 8A) unresolvable in LM (Fig 4C–4F). In CPD-SEM preparations, some of the net components appeared as large scales with rays radiating out from a central annulus. The webs and nets were mostly non-siliceous (Figs 6A, 6B, 8B and 8C), silicification areas near the EDS detection limit could be found where webs were interconnecting and in some of the annuli. On the underside of the shrouds, near the surface of the spherical auxospores, we found a layer of smaller, delicate dendritic scales. Some of the dendritic scales had minute annuli, but others originated from a fractal core (Figs 6D and 7A). Layers of these dendritic scales densely covered spherical and small anisodiametric auxospores, but they were also found on the surface of larger ones and a few were often scattered even on mature auxospores (Figs 8D and 9A).

More typical, strongly silicified incunabular elements (scales) came in a variety of sizes. Examples of disarticulated elements are shown in Fig 6E. They occurred underneath the shrouds and dendritic scales and were not restricted to covering the auxospores in their spherical stage of development. Instead, when exposed by gentle chemical cleaning, such elements could be seen all over the surface of anisodiametric auxospores of all sizes. Transition from incunabular to the other wall-layers was often unclear in SEM preparations even if the deposition of silica in distinct, separate and originally physically distant layers was apparent in PDMPO-labeled walls. Note multiple separate layers of PDMPO-tracked silicification in Figs 4D, 4F, 4J, and 5H and compare to Fig 6E.

#### Epizonium and perizonium

Other strongly silicified components of the wall were organized in four structurally different layers: two layers of epizonium (outer and inner) and two types of perizonium (TP and LP).

**Fig 10:**
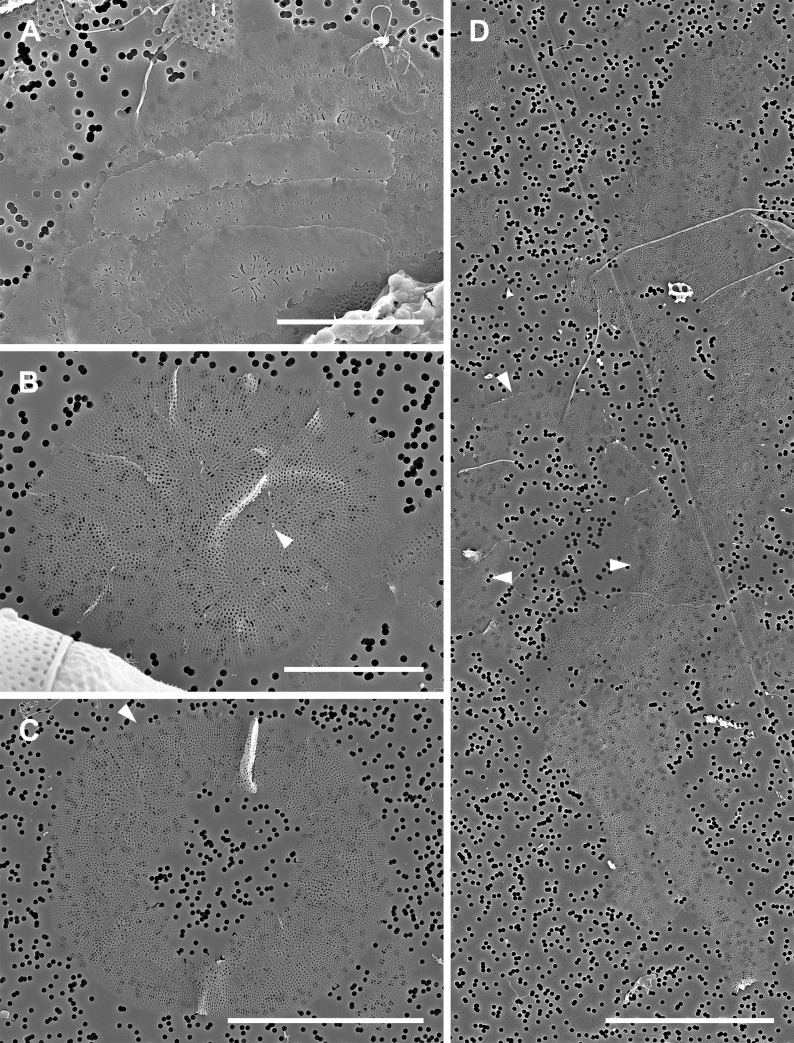
Siliceous elements of the auxospore walls (SEM, acid cleaned specimens). (A) An aggregate of detached elements of the incunabulum and/or outer epizonium, elongated scales and bands illustrating diversity of size, shape, and ornamentation pattern. (B) A large scale of the inner epizonium with four separate patterning centers (one indicated by arrowhead), possibly a result of fusion of multiple smaller scales (C) Circular TP band from near auxospore apex. Note that it also consists of a number of coalesced subunits, one junction indicated by arrowhead. (D) Several TP bands consisting of fused shorter elements. Note fragments of a subapical ring-band (arrowheads) at the left center. Digital montage of 2 separate images. Scale bars: A, B, 20 μm; C, D, 40 μm.

Epizonium consists of “the layers of irregular, unsystematically wound, coarse bands located between scales and much more symmetrical perizonium” ([2]; p. 128, 132), and perhaps the structures covering the poles in angular auxospores, such as *Lithodesmium* ([2]; p. 140). In our auxospores, the epizonium was strongly developed and complex. It contained two layers made of structures with different form and ornamentation. The external layer (outer epizonium) consisted of strongly silicified, nearly solid scales and bands of various lengths and perforation patterns, including irregularly shaped and/or branching forms (Figs 6E, 9A, 9B and 10A). Some of the longest bands showed a degree of systematic oblique orientation relative to the apical length of the auxospore (Fig 9B). A slightly concentric organization of bands near the apices suggesting that they may be wrapping around them can also be seen in this image. Underneath this layer, there was a layer of large but more delicate circular and broadly ellipsoidal scales and short bands densely ornamented by pores arranged in a regular radial pattern; the inner epizonium (Figs 6E, 9C and 10B). Particularly notable were large scales found around the auxospore apices (Figs 7B, 8A, 9A and 9C) forming apical caps under the incunabular layers on mid-sized and large auxospores.

**Fig 11:**
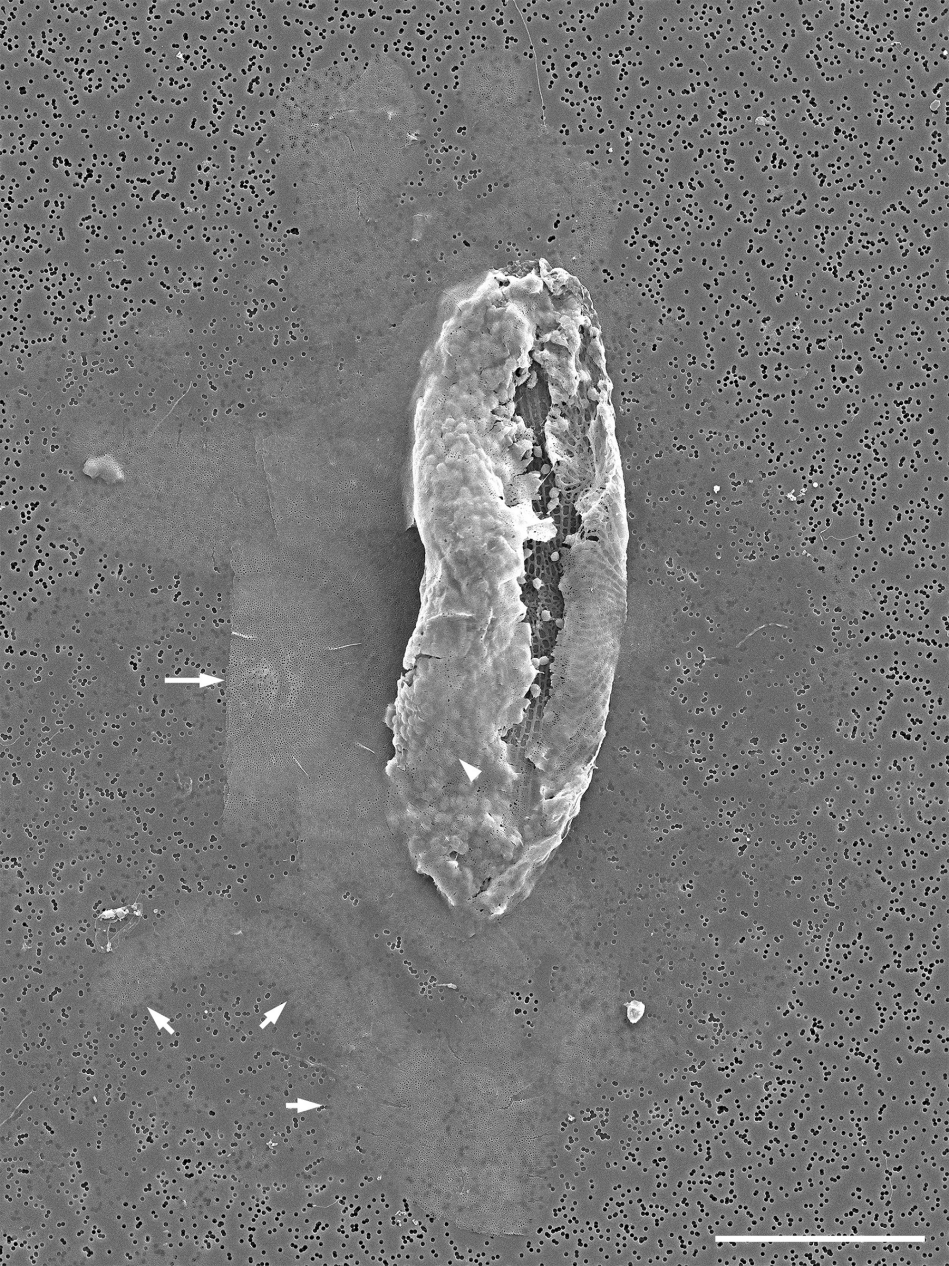
Partially disarticulated, nearly mature auxospore (SEM, acid cleaned specimen). Mature auxospore harboring basal silica layer of the initial epivalve (right side). Note deepest layers of auxospore wall have been exposed, showing TP (primary band indicated by long arrow), one of the LP bands (arrowhead) and subapical rings of TP bands (short arrows). Scale bar: 50 μm.

**Fig 12:**
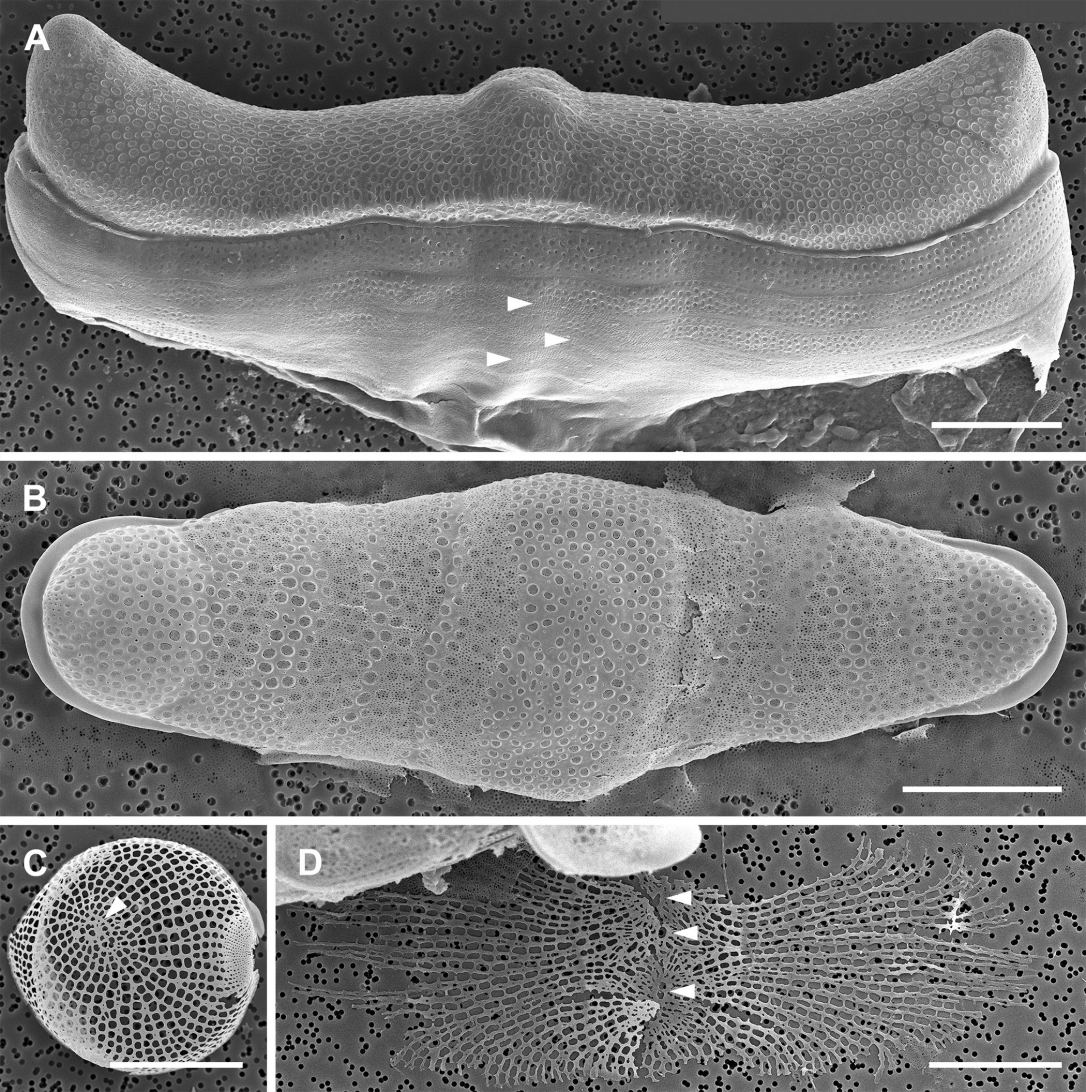
Parental and initial valves (SEM, acid cleaned specimens). (A) External view of auxospore with initial epitheca and partially damaged bottom side of auxospore wall. Remnants of TP bands visible near the left apex while LP bands are best seen below central elevation of the valve (arrowheads). Digital montage of two separate images. (B) Initial valve swathed by remains of disarticulated TP. Note they are still nearly perpendicular to the valve apical axis near valve mid-section while slightly arching towards the apices near valve ends. Digital montage of two separate images. (C) A representative valve from the population of parental cells at early stage of morphogenesis showing valve face with one (?) annulus (arrowhead) and one elevated apical pore field. (D) Basal silica layer of a large postsexual valve showing three small annuli (arrowheads). All scale bars: 25 μm.

The perizonium was also complex and multilayered. The innermost parts of the auxospore wall consisted of bands of LP and TP (Figs 10C, 10D, 11, 12A and 12B). The deposition of the primary TP band became evident when the auxospore center attained a slim and rigid appearance. In our material it occurred when cells were approximately 100–200 μm in apical and 50–100 μm in transapical axis (Figs 4K, 4L and 11). The primary TP band was open, widely elliptical, and shorter than others (Fig 11). The secondary and the next closest consecutive band may have been closed and encircling the primary band, although this remains unclear. Primary and other TP bands carried similar ornamentation (Fig 11). They consisted of a string of “units”, each perforated by rows of pores radiating from its own pattern center. Individual “units” were similar to what were seen as individual scales. Several such pattern centers could be seen on each band (Fig 10C and 10D). Altogether, TP band structure and perforation pattern gave the impression that the bands originated from fusion of a string of scales or short bands made of such a string of scales, thus the name “scaly-bands”. Junctions of these sub-units are indicated by arrowheads in Fig 10C and 10D. Secondary TP bands were of approximately equal width and carried rows of pores and fimbria on both sides of a perforated mid-section. TP bands located over the middle of the initial valve face slightly slanted toward the primary band while the others were perpendicular to the apical axis of the auxospore (Fig 12B). Their ends aligned, but did not usually meet, leaving an area free of bands on the ventral surface of the auxospore (Figs 4H, 8A and 9A). However, bands closer to the apices did meet, as also could be seen traced with PDMPO (Figs 4H, 8A and 8D). Apical TP bands formed rings arching slightly towards the apices (Figs 10C and 11). Longitudinal perizonium (LP) was also present. Its bands were longer and narrower than the transverse type, but their ornamentation was similar (Figs 11 and 12A). In mature auxospores some of the previously deposited wall elements were either shed off (halos) or thinned and appressed together, with remains stretching over the developing initial valves and thus obscuring its complex, multi-layered nature (Fig 8D). Both outer epizonium and TP showed bands in an overall similar configuration: concentric bands near apices, slanted/perpendicular at the cell mid-section. The outer epizonium was more solidly structured and ornamented than TP. On the other hand, structural constituents of the inner epizonium and TP bands were similar in their ornamentation but differed in form and organization on the auxospore surface. It also ought to be considered that the outer epizonial layers in our clone represent an exceptionally elaborate incunabulum, while the inner epizonium could be a part of an unusual perizonium. More research is needed before the nature of these layers is fully understood.

#### Initial frustules

Three types of initial valves were found in the same mating dishes. The most common were oblong valves with parallel, slightly crenate margins (160–300 μm in apical and 40–107 μm in transapical axes; Figs 5E, 5F, 8D and 12A). A few valves had a notable central swelling separating the mid-section from the apices (Fig 12B). Finally, exceptionally rarely, initial frustules with tripolar valves were also present. Valve apical length comparison between the gametangial and initial valves indicated that the initial cells could be 3–12 times longer than their parents, but most commonly were only 5–6 times longer. A representative valve of a parent is shown for comparison in Fig 12C.

Overall fine morphology of initial valves was different from normal vegetative valves, more so in the case of the initial epivalve than the initial hypovalve. The apical elevations of initial epivalves were lower and less differentiated and apical pseudocelli were less defined than on normal vegetative valves (Fig 12A and 12B). The internal costae were more disorganized on the initial epivalve compared to those on initial hypovalves or typical, full size vegetative valves. Moreover, initial, and immediately postsexual valves possessed two or more small and irregular annuli (Fig 12D). The annuli were most visible before the basal silica layer was obscured by valve superstructures added later. Subsequent postsexual valves carried better developed annuli and normally only one or two per valve face, on or near small mid-valve umbonate elevations. In bi-annular valves, the number of annuli likely decreases to one as the valves diminish, similar to what can be seen on small parental valves (Fig 12C). We encountered no processes of any kind on parental, initial or postsexual valves. Areolae (5–6 rows in 10 μm) and scattered pores perforated the valve faces. Areolae were occluded by elaborately branched vela and had rimmed borders. The initial mantle was very narrow with an irregular free margin. Initial valvocopulae did not fuse with the valve mantle. All copulae were perforated by rows of pore areolae (Fig 12A). The trans-valvar axes of the initial frustules recovered were 50–73 μm long and consisted of up to 9 copulae associated with the initial epivalve.

## Discussion

### Clone identity

The diatom *Biddulphia biddulphiana* is reported worldwide, but the name itself has a considerable history of taxonomic confusion. Well over a hundred years ago, several morphologically similar species were described independently and given different names, many of which were later synonymized. Two of the most consistently mentioned conspecifics were *B*. *biddulphiana* [29] and *B*. *pulchella* Gray [34], with different authors giving priority to one or the other name (compare [1, 22, 29]). This ambiguity was likely driven by the broad diagnoses, sometimes focused on a single specimen. In contrast, well documented specimens of *B*. *biddulphiana* and *B*. *pulchella* exhibit a range of morphometric characters. For example, the specimens shown in Schmidt ([21]; pl. 118, figs 25–32, pl. 121, figs 1–2, completed in 1888 and named *B*. *pulchella*) are broadly elliptical in valve face view and have sinuate margins. Nearly all show “spines” (likely representing rimoportulae and/or occluded processes; pl. 118, figs 28, 29). This type of sinuated, stocky elliptical valve morphotype and rimoportula arrangement has also been documented using SEM [12, 30, 31, 32]. Hustedt [22], on the other hand, presented a somewhat different valve morphotype, with a more oblong than oval/elliptical valve in face view and gently crenated instead of sinuated margins. However, they all either illustrate or describe a single oblong or panduriform annulus. Both sinuate and crenate morphotypes conform to the original description metrics and both exhibit valve processes. The causative factors and/or conditions responsible for transitions between these morphotypes, if they occur, remain unknown.

Characters of the valves of our clone differ from the two morphotypes discussed above. Most apparent is the absence of valve processes on any of the valves examined, whether initial, postsexual, large, or small vegetative types. In *B*. *tridens*, the sister taxon to *B*. *biddulphiana*, rimoportulae form late in valve morphogenesis, near the inner rims of the annulus when the internal structure of the annulus begins to develop [35]. Thus, if also true for our diatom, mutation in the annulus development could negatively affect the development of the associated processes. As seen from the results, if such mutation occurred, it is heritable in our clone’s homothallic mating. Individuals and clones with crenate margins and no processes are known from other, mostly Atlantic sites. They are discussed in greater detail by Hoban [12]. The number of annuli on the initial and postsexual valves also differ from initial valves documented for this taxon as currently defined, albeit in a very few studies. Neither initial nor postsexual valves develop a panduriform [29, 30] or a transversely oblong annulus reported in previous studies [22, 29, 31] for large vegetative valves. Instead, our initial valves have 2–4 small, circular annuli, while early postsexual valves have mostly two (occasionally one). It may also be expected that valve proportions and outline may change, and ornamentation details simplify over mitotic generations because of “diatom cell diminution” [36, 37; p. 83, 38, 39]. Consequently, large individuals of closely related species will likely be morphologically more different from each other (showing more sophisticated morphologies) than when they become small (outlines likely simplified). Thus, the specimens closer to postsexual cell-size may be the most informative in morphological comparisons of previously synonymized taxa. In this context and given that the clone examined here meets only some of the characters of either of the two morphotypes commonly attributed to *B*. *biddulphiana*, we provide illustrative documentation of both the parents and progeny for future taxonomic reappraisal.

### Sexualization and gametogenesis

Both oogonia and spermatogonia were observed in our experiments, in far greater quantities after induction than when sexualization was spontaneous. In both cases the clone behaves as a homothallic, probably inconstant, self-fertile hermaphrodite [25]. This is consistent with observations of von Stosch [2] and similar to reproductive behavior in the sister species, *B*. *tridens* [16].

Gametogenesis and gamete structure are also consistent with the earlier, although basic descriptions [2, 12]. In our (Florida) and Roscoff [2] diatoms, oogenesis results in one egg per oogonium, while two semi-intercalary eggs were produced by parents isolated from the Texan coast of the Gulf of Mexico [12]. Species producing either one or two eggs have been reported in various polar diatoms [25, 40], so oogenesis in our clone is of a common type.

In contrast to oogenesis, spermatogenesis in our clone is thus far unique. It involves unequal cytokinesis of the spermatocyte mother cell (thus before meiosis) resulting in separation of a non-sexual residual body from a colorless primary spermatocyte. This differs from spermatogenesis in other diatoms where residual bodies are produced during or after meiosis and not before (see [25]; Fig 7, [41]). Residual bodies produced during gametogenesis have been previously documented in a number of centric species and a few araphid pennates [25]. The process is more common in spermatogenesis than oogenesis. Two primary spermatocytes (and two residual bodies) are produced by our spermatogonangia in contrast to just one produced by Roscoff individuals [2]. It is unfortunate that neither Roscoff nor our samples revealed information regarding sperm flagella. Flagellated sperm presence is nonetheless presumed because numerous open spermatogonangial thecae without spermatids but containing their chloroplast-filled residual bodies were found in our mating dishes. No information regarding the Texan clones is available. Comparatively higher fecundity in both sexes was observed in the sister species, *B*. *tridens* [16]. There, spermatogonia of similar size produced up to 16 primary spermatocytes. Thus, the unique process of sperm production seen in our and the Roscoff specimens supports the notion that diatom spermatogenesis is one of the most variable aspects of their sexual reproduction and may differ significantly even between relatively closely related taxa ([42]; fig S1).

### Auxospore structure and development

Auxospore development follows the three stages expected in polar diatoms: isodiametric, anisodiametric and deposition of initial frustules [42]. All previously known structural contributors to the auxospore walls (incunabula, epizonia, TP and LP) are present. The greatest difference in the wall structure found here compared to other polar diatoms involves the relative abundance, diversity, and persistence of some of them. Incunabular and epizonial elements are most diverse. The growth pattern however has not been previously documented. It includes asynchronous outgrowth of the apices, persisting incunabula and extensive layers of epizonium covering the entire auxospore, deposited before and in addition to typical perizonia. Here, we resurrect the term “epizonium” for our auxospore wall layers between incunabulum and perizonium when such exist, even though we concur with the reasoning in Kaczmarska et al. [25] which will apply to most other species thus far examined.

Novel to the auxospore wall of centric diatoms are thick incunabular shrouds that, at best, are very lightly silicified. Despite their delicate appearance, many persist throughout the duration of cell growth. The richly organic nature of the incunabular shrouds found here undoubtedly contribute to the capacity of the wall to stretch and maintain envelopment of various siliceous elements around the growing protoplast. Incunabular walls so well endowed with organic material are thus far known only among a few core araphid pennates, such as two species from the genus *Rhabdonema* and some tabellarioids ([2]; p. 133). Presumably entirely organic investments that thickly surround developing auxospores (copulation envelopes; [25]) are more frequent among the raphid pennates, where their main function is believed to hold mating partners in a position favorable for fertilization rather than to protect developing auxospores. Neither the majority of polar centric nor the thus far investigated members of the basal araphid auxospores produce lasting or thick incunabular layers [39], rendering the incunabular shroud in our clone specific to this diatom.

In addition to organic components, we found more typical, well silicified elements with various ornamentations, shapes, and sizes. Their ornamentation varies from radial to pinnate patterns. The shapes vary from circular scales to straps, bands, irregular and branching structures and occur in a range of sizes. Similar elements are known from incunabula and perizonia of other mediophycean diatoms, but not in such diversity in a single wall. Exactly how and when incunabula transition into the epizonial part of the wall and this in turn into the perizonial part remains unclear because neither of the older wall elements rupture to expose younger, newly deposited layers, as seen in most diatoms with polar valve outline, both centric and pennate, e.g., *Chaetoceros* spp., *Ardissonea crystallina* (C. Agardh) Grunow, *Craticula cuspidata* (Kutzing) D.G. Mann [2, 37, 42]. Instead, all layers stretch (or new elements added) to cover the growing auxospore until the initial frustule is formed, thereby obscuring the later deposited layers.

Relatively little is known about epizonia beyond a few images from some mediophycean species discussed by von Stosch [2]. Their role in shaping auxospores is poorly understood, but their form and position suggest involvement in shaping/protecting corners of some multipolar auxospores ([2]; p. 140). In our auxospores, epizonial layers are extensive, differentiated into outer and inner types and cover the entire auxospore. Large auxospores suggest concurrent silica deposition in more than two regions: the two apices and in the cell mid-section. If correct, this process differs from most commonly seem TP deposition sequences in mediophyceans and pennates. In mediophyceans, the TP bands are deposited concentrically and progressively away from the primary band (*Attheya*, *Lithodesmium*, *Odontella*; [2]). Similarly, in pennates, TP deposition is typically bidirectional such that new bands are also added progressively away and parallel from the primary band [37].

Although some epizonial elements from *B*. *biddulphiana* were already shown by von Stosch ([2]; bands) and Hoban ([12]; scales), the abundance and variety of these structures is shown here for the first time. Compared to Roscoff specimens, our auxospores show less symmetrically organized bands of outer epizonium, while compared to Texan individuals, scales of the inner epizonium are less robust. Nonetheless, we observe that even the scaly apical caps of the inner epizonium are already present in lozenge-shaped midsize auxospores, possibly before the first perizonial bands develop. Therefore, it is plausible that the epizonia, particularly the outer epizonium (developing before the inner epizonium), participate in shaping the earliest stages of anisodiametric growth in our clone. Using the type of ornamentation of the TP bands as a guide, we observe that the inner epizonial layer shows ornamentation similar to that seen on the perizonium, while ornamentation of the outer epizonial elements is much more diverse. Some are similar to incunabular scales [16] and straps (see vincula in *Ardissonea*; [43]), but others are comparable to pinnate bands in the TP of pennate diatoms (*Rhoicosphenia*; [44], *Gephyria*; [45], *Grammatophora*; [46]). In eupodiscacean species, the epizonium may be absent (*Odontella aurita* (Lyngbye) C. Agardh) or present only at the auxospore apices (*Trieres regia* (M. Schultze) Ashworth & E.C. Theriot). In both cases however, the forms are different from that observed in our clone and from each other [2].

The structure and ornamentation of perizonia in our clone is similar to that found in other mediophyceans. Here too, they show affinity to scales in terms of ornamentation pattern, but in many cases, it can be clearly seen that parts or entire bands form through anastomosis of a string of initially separate scales, thus the name “scaly-bands”. This is consistent with the majority of mediophyte species examined to date using SEM and contrast with the pennate bands which typically have a pinnate ornamentation (with a strong median rib and fimbria). Both open and closed TP bands are found in our clone. One or two TP bands surrounding the primary TP might have been closed. Other bands are open and in mature auxospores oriented nearly perpendicularly to the initial valve apical axis. They tend to align their free ends. Closed bands are again found around the conical auxospore apices. This contrasts to the TP bands “fanning out” or looping around auxospores seen in other mediophytes, such as Chaetocerotales, *Attheya* or Lithodesmiales. *Biddulphia tridens* TP band structure is similar to that found in *B*. *biddulphiana*, but *in situ* configuration for all of them remains unclear because the auxospore wall siliceous elements are delicate and layers difficult to separate without damage. In the eupodiscacean species examined, TP bands show a stronger non-perpendicular configuration and non-aligned ends [2]. Among other mediophyte species, TP bands oriented perpendicularly to the auxospore apical axis are known in only one species, the exceptionally long bipolar centric diatom *Ardissonea crystallina* [42, 43]. Open bands with aligned ends and perpendicular orientation relative to the cell apical axis are typical of pennate auxospores [2, 42]. Thus, the perpendicular and open TP bands with aligning ends in our auxospores are characters shared with TP of the majority of pennates.

Initial and postsexual valves produced by our clone share some characters (valve topography, size, and areolar density) with those documented for *B*. *biddulphiana* from the Texan Gulf of Mexico [12] and the Californian shore of the Pacific Ocean [30]. Similar to many of our initial valves, Texan specimens show disorganized internal costa and the absence of any processes on all valves. The form of their annuli on initial valves is unknown. Our initial valves carry more differences compared to the natural population from California. A single initial valve illustrated from the inner side shows no internal costa, has rimoportulae, and one large, oblong annulus. However, in the sympatric population of typical vegetative valves from which parents of the initial cell were likely derived, only one panduriform annulus per valve was illustrated on all valves presented.

### Comments on evolutionary relationships

Persistent, thick, richly organic incunabula and parallel, open TP bands with aligning ends make *B*. *biddulphiana* auxospores similar to the few araphid pennates that were available for comparison in the early 1980s [2]. Based on then available data, von Stosch hypothesized a closer evolutionary relationship between araphid pennates and members of the family Biddulphiaceae when compared to the Eupodiscaceae that was suggested by Simonsen [5].

However, it has since become apparent that thick and persisting incunabula are infrequent among araphid pennates, albeit the number of araphid species whose sexual reproduction and auxospore structure has been examined remains disappointingly small. Such extensive incunabula also cannot be found in clones of the *B*. *biddulphiana* sister species *B*. *tridens* or in any of the basal araphid pennates [16, 19, 39, 47]. There are a few characters in TP band arrangement and structure common in those that are shared with various groups of diatoms with polar valve outline. The fanning-out of closed TP bands encircling the primary TP band is one of the most frequent among mediophytes, particularly those with bipolar valves. However, a somewhat similar configuration is also known among a few basal and non-basal araphid pennates, e.g., *Gephyria*, *Plagiogramma*, *Dimeregramma*, and *Grammatophora* [39, 45, 46]. Neither the eupodiscacean species nor our clone show closed TP bands with a fanning-out configuration. As in our clone and some eupodiscacean species, TP also include rings and/or loops slanting towards the apices. In our clone, the mid-section set of TP consists of open bands perpendicular to the apical cell axis and parallel to each other. Outside of the auxospore mid-section, some bands are not perpendicular to its apical axis or are not open. However, those that are open have their free ends aligned across a ventral space in between, and the same may be inferred from photographs of specimens from Roscoff [2]. These three characters (bands perpendicular, open, ends aligned) are shared with the majority of pennate TP bands. Thus, *B*. *biddulphiana* TP bands possess a mixture of characters. Some are known from among mediophyceans (bands scaly, closed rings), while others are typical of pennates. Nonetheless, the overall development and structure of our auxospores conform to those of mediophyceans. The auxospores of the exceptionally long diatom *Ardissonea crystallina* [43] are more similar in overall morphology to pennate auxospores than are those of our clone or any known Eupodiscacean.

One of the species-specific features of our auxospore wall structure, the epizonium, emerges as interesting and potentially evolutionarily informative when considered in a broad context. Multiple layers of epizonium illustrate an unanticipated diversity of size, form and ornamentation of siliceous elements that can be harbored in a single auxospore wall. The diversity bridges differences between incunabula and perizonia in mediophytes and may illustrate modifications that some of the inner layers of simple small scales in the ancestral auxospore wall might have undergone in their transformation into perizonia, perhaps of more than one type. We note that the walls of the coscinodiscophycean auxospores normally consist of several layers of incunabular scales and some variation in size and shape already exist in all that have been examined.

The function of the epizonium is presently unclear. Von Stosch [2] suggested that epizonia may be involved in shaping corners of angular auxospores such as *Lithodesmium* and possibly other species within this and other genera. However, in our clone, the epizonium covers the entire auxospore and appears in the early anisodiametric stage of development, thus before perizonia are formed. In the auxospore walls of many mediophytes, epizonia are absent or diminished in abundance and diversity, but happen to persist in our clone. Currently, there is no clear evidence that auxospores of non-polar centrics or pennates carry epizonia.

In summary, what is currently known about auxospores in species with biddulphioid frustules (mostly members of Eupodiscaceae) demonstrate that their ontogeny and wall structure are different in nearly each species examined and are different from the two species of *Biddulphia* examined to date. In the latter, details differ also between clones isolated from different geographies. Sadly, both the number of species and clones investigated remains very small, resulting in nearly every study bringing to light novel, unanticipated aspects of diatom sexuality and auxospore development, yet yielding no clear indication which group of mediophytes harbors the elusive closest currently living relative of pennates.

## Supporting information

S1 Appendix Spreadsheet with values from which measurement ranges were derived. (XLSX)
